# Role of the oral microbiota in cancer evolution and progression

**DOI:** 10.1002/cam4.3206

**Published:** 2020-07-07

**Authors:** Jiwei Sun, Qingming Tang, Shaoling Yu, Mengru Xie, Yanling Xie, Guangjin Chen, Lili Chen

**Affiliations:** ^1^ Department of Stomatology Tongji Medical College Union Hospital Huazhong University of Science and Technology Wuhan China; ^2^ Hubei Province Key Laboratory of Oral and Maxillofacial Development and Regeneration Wuhan China

**Keywords:** cancer, carcinogenesis, infection, oral microbiota

## Abstract

Bacteria identified in the oral cavity are highly complicated. They include approximately 1000 species with a diverse variety of commensal microbes that play crucial roles in the health status of individuals. Epidemiological studies related to molecular pathology have revealed that there is a close relationship between oral microbiota and tumor occurrence. Oral microbiota has attracted considerable attention for its role in in‐situ or distant tumor progression. Anaerobic oral bacteria with potential pathogenic abilities, especially *Fusobacterium nucleatum* and *Porphyromonas gingivalis*, are well studied and have close relationships with various types of carcinomas. Some aerobic bacteria such as *Parvimonas* are also linked to tumorigenesis. Moreover, human papillomavirus, oral fungi, and parasites are closely associated with oropharyngeal carcinoma. Microbial dysbiosis, colonization, and translocation of oral microbiota are necessary for implementation of carcinogenic functions. Various underlying mechanisms of oral microbiota‐induced carcinogenesis have been reported including excessive inflammatory reaction, immunosuppression of host, promotion of malignant transformation, antiapoptotic activity, and secretion of carcinogens. In this review, we have systemically described the impact of oral microbial abnormalities on carcinogenesis and the future directions in this field for bringing in new ideas for effective prevention of tumors.

## Graphical abstract

Oral microbiota has been playing an important role in the development of cancer throughout human body. Thus we try to summarize the current researches about this topic.

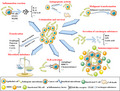

## 1 INTRODUCTION

Currently, cancer is the most disturbing disease in humans, affecting almost all regions in the body. Many factors such as chemicals, radiation, and physical agents are associated with its occurrence.^1^ Among all the causes of cancer, role of microbes had been disregarded for a long time until landmark studies in the early 1990s established *Helicobacter pylori* (*H. Pylori*) as a causative agent of gastric cancer, resulting in a paradigm shift in implicating infectious agents as meaningful contributors to the process of gastric cancer.^2^ Subsequent studies placed the worldwide risk of cancers from microbial infections at 16.1%, further implicating the role of microbiota.^3^ The number of human cancers associated with viruses has grown. Human papillomavirus (HPV) is linked to cervical carcinoma, Epstein‐Barr virus (EBV) is linked to nasopharyngeal carcinoma, and hepatitis B virus (HBV) is linked to hepatocellular carcinoma.^4^ The most classical example of bacterial tumor induction is the induction of gastric cancer by *H. Pylori* through host‐microbial interaction.^5^ *Schistosoma haematobium* is closely associated with bladder squamous cell carcinoma^6^ and *Opisthorchis viverrini* is linked to cholangiocarcinoma.^7^ Treatments aimed at decreasing the infectious damage seem to improve the prognosis of infection‐associated cancers. A recent study has proven that eradication of *H. Pylori* seems to counteract the development of gastric adenocarcinoma.^8^ Actinomycin C and D and mitomycin C have been recognized for their antitumor effect.^9^ These studies have proven the role of microbial dysbiosis in cancer progression. According to the updated researches, a large group of tumor‐associated microbiota is implicated in tumorigenesis, while a small number of microbes have an antitumor effect. The recent isolation of a group of 11 commensal bacterial strains from gut microbiota has been shown to manipulate the anticancer immunity of the host.^10^ Even before this discovery, isolation of *Streptococcus pyogenes* from neck cancer had laid out the groundwork for potential use of microbiota as anticancer agents.^11^ Many animal models have been established to confirm the anticancer role of these microbes.^12^, ^13^, ^14^ This phenomenon indicates that the interaction between microbiota and cancer is a complex one with some of the microorganisms showing tumorigenic effect and others exhibiting anticancer function.

The oral cavity harbors a huge collection of microbes including bacteria, fungi, viruses, and bacteriophages and is considered one of the greatest microbiological reservoirs in human body.^15^ Some well‐studied periodontal organisms have now emerged as focal points for the developing association between oral microbial dysbiosis and cancer.^16^ *Fusobacterium nucleatum* (*F. nucleatum*) from the oral cavity is considered a pivotal promoting factor for colorectal cancer (CRC). Moreover, periodontal health status has been confirmed to be associated with esophageal cancer and pancreatic cancer. Microorganisms from other parts of the body have been identified to play a role in tumor progression and chemoresistance.^17^ These correlations between oral microbiota and carcinogenesis have shed some light on the ongoing explorations in this field, confirming the opinion that oral microbiota might contribute significantly to tumorigenesis through multiple mechanisms and a deep understanding of these mechanisms might help in resisting tumor progression. Correlation of oral microbiota with throat cancer and pancreatic cancer has been elucidated by Wang et al and Karpinsky.^18^, ^19^ Impact of oral microbiota on distant tumors was reported by Mascitti et al.^20^ Underlying roles of oral microbiota as effective clinical biomarkers for the diagnosis of gastrointestinal carcinomas were summarized by Zhang et al and Chen et al.^21^, ^22^ Preliminary oral microbial carcinogenic mechanisms were summarized by Karpinski.^23^ Based on the existing reviews focusing on this topic, we performed a systematic review of the existing studies, updates in the knowledge about this field, and the current research progress.

## 2 A COHORT OF ORAL MICROBIOTA WITH A STRONG IMPACT ON CARCINOGENESIS

**FIGURE 1:**
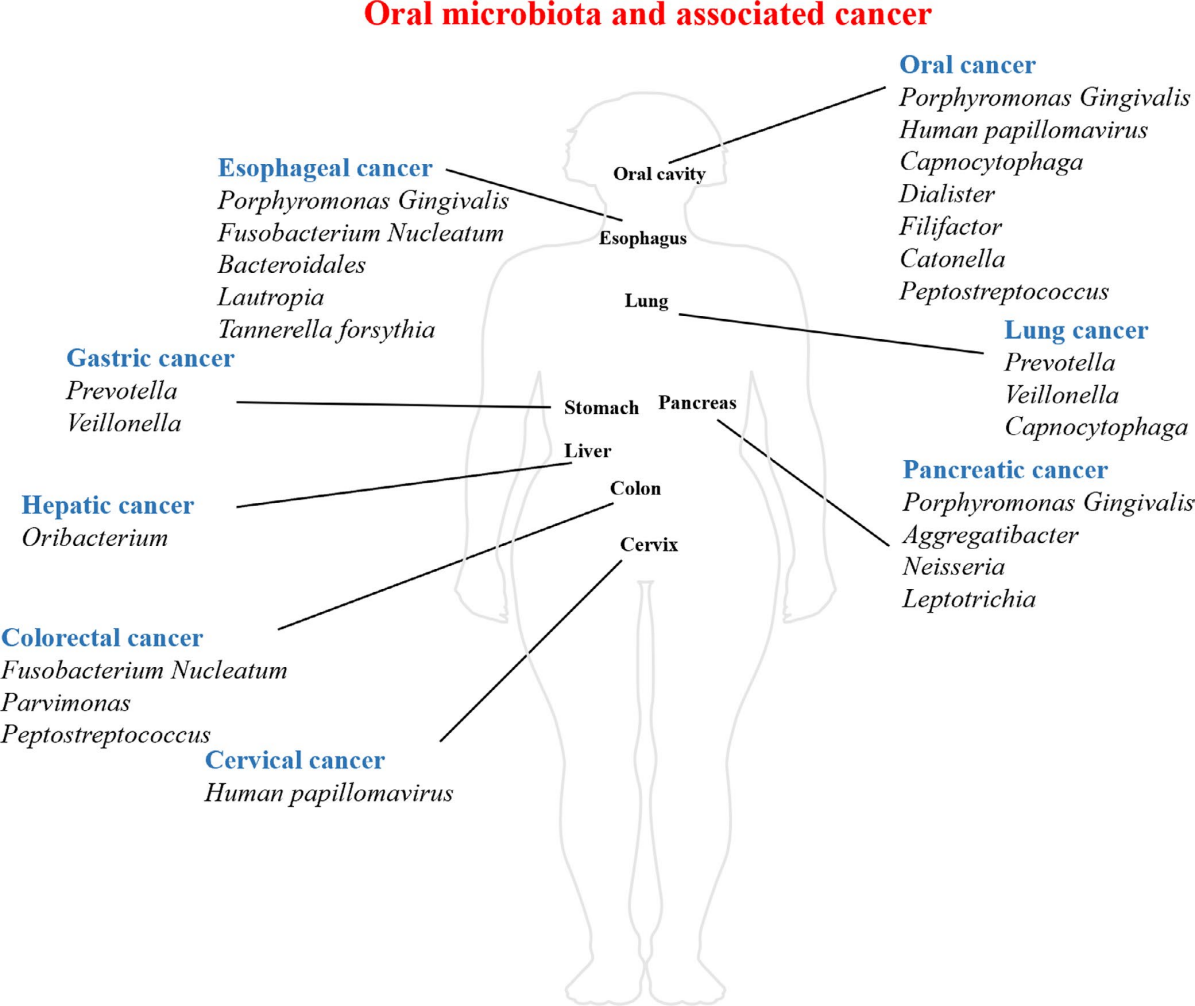
Distribution of oral microbiota and associated cancer. This figure describes the distribution of oral microbiota through human body, and their influence on certain types of cancers. Besides oral cavity, esophagus, pancreas, colon, lung, liver, stomach as well as cervix are also correlated with spread of oral microbiota

Currently, methods have been developed to evaluate and decide the status of infection. These advances have helped build the association between oral microbiota and tumorigenesis. A considerable number of oral bacteria have been linked to carcinogenesis. Among them, *F. nucleatum* and *Porphyromonas gingivalis* (*P. gingivalis*) have been implicated in the progression of various carcinomas. Some of the other anaerobic and aerobic bacteria have also been reported to show a correlation with carcinogenesis. The role of HPV in the progression of oropharyngeal carcinoma has been widely acknowledged. Abnormalities related to oral fungi and parasites might also be associated with carcinogenesis, though the evidence regarding their role is lacking (Figure 1; Tables 1 and 2).

**TABLE 1:** Detection of certain oral microbiota in cancerous sites

Detecting method	Associated cancer type	Sample type	Microbiome	References
16S rRNA sequencing	Oral squamous cell carcinoma	Tissues	*Actinomyces* *Parvimonas* *Fusobacterium nucleatum* *Pseudomonas aeruginosa* *Dialister*	24, 25, 26, 27, 28, 29, 30, 31, 32, 33, 34, 35
16S rDNA sequencing	Pancreatic cancer	Saliva	*Porphyromonas gingivalis* *Aggregatibacter Actinomycetemcomitans* *Fusobacterium* *Streptococcus* *Neisseria* *Capnocytophaga*	
	Lung cancer	Saliva	*Capnocytophaga* *Veillonella*	
	Throat cancer	Saliva	*Aggregatibacter* *Pseudomonas* *Bacteroides* *Ruminiclostridium*	
	Gastric cancer	Saliva	*Streptococcus* *Prevotella* *Prevotella7* *Veillonella*	
Metagenomic sequencing	Colorectal cancer	Fecal	*Fusobacterium*	36, 37
	Esophageal cancer	Saliva	*Streptococcus pneumoniae*	
Pan‐pathogen array	Oral squamous cell carcinoma	Tissues	*Bacteria*, *viruses*, *fungi*, etc	38
qPCR	Colorectal cancer	Tissues	*F. nucleatum*	37, 39, 40
	Hepatic cancer	Saliva	*Oribacterium* *Clostridium* sp.	
	Oral carcinoma	Saliva	*Enterobacteriaceae*	
Immunological staining	Esophageal cancer	Tissues	*P. gingivalis*	41, 42
	Oral squamous cell carcinoma	Tissues	*P. gingivalis*	
Antibody detection	Esophageal cancer	Serum	*P. gingivalis*	43, 44, 45
	Pancreatic cancer	Serum	*P. gingivalis*	
Fluorescence in‐situ hybridization	Colorectal cancer	Tissues	*F. nucleatum*	46, 47

**TABLE 2:** Impact of oral microbiota on carcinogenesis

Microbiome	Type	Associated cancer	Spreading passage	Effect	References
*Fusobacterium nucleatum*	Gram negative	Colorectal cancer	Digestive tract	Promoting	24, 25, 26, 27, 36, 37, 39, 40, 46, 47, 48, 49, 50, 51, 52, 53, 54, 55, 56
		Esophageal cancer			
*Porphyromonas gingivalis*	Gram negative	Oral squamous cell cancer	In‐situ oral cavity	Promoting	41, 42, 43, 44, 45, 57, 58, 59, 60, 61, 62
		Pancreatic cancer	Blood stream		
		Esophageal cancer	Digestive tract		
*Human papillomavirus*	DNA virus	Cervical cancer	Sexual behavior	Promoting	63, 64, 65, 66, 67, 68, 69, 70, 71, 72, 73, 74
		Oral cancer			
		Head and neck cancer			

### 2.1 *Fusobacterium nucleatum*

*Fusobacterium* is a Gram‐negative anaerobic bacillus mainly located in the oral cavity and in the gastrointestinal tract. It is a natural inhabitant of the oral cavity. However, *Fusobacterium* has long been considered an opportunistic infectious agent in humans.^48^ During in‐situ oral squamous cell carcinoma (OSCC), bioinformatic prediction analysis of sequencing data indicated that a high abundance of *F. nucleatum* subspecies *Polymorphum* was found in oral cancer tissues.^24^ *F. nucleatum* has recently been implicated in CRC.^49^ A higher relative abundance of *Fusobacterium* in CRC samples was identified by 16S rRNA sequencing.^25^ Increased *Fusobacterium* levels in stools of CRC patients were detected using whole‐genome shotgun sequencing.^36^ Fecal samples from other cohorts were also studied using metagenomic sequencing to confirm the presence of *Fusobacterium* and its species, suggesting that microbial signatures could be used as non‐invasive biomarkers.^37^ The presence of *F. nucleatum* was validated after sequencing using quantitative polymerase chain reaction (qPCR) to detect its specific gene markers.^37^ With *F. nucleatum*‐specific primer sets, it was confirmed that *F. nucleatum* detected in the CRC samples showed a high similarity with that detected in the saliva,^39^ indicating the possible oral origin of *F. nucleatum* in CRC samples. With the help of fluorescence in‐situ hybridization technique, *F. nucleatum*‐specific fluorescent probe was used to reveal the significant abundance of *F. nucleatum* in colorectal tumors.^46^ The invasion of *F. nucleatum* into CRC cells was also demonstrated with the help of fluorescent probe.^47^ Patients with cancerous tissues having higher loads of *F. nucleatum* DNA had shorter survival durations,^50^ making it a potential biomarker for prognosis. Studies have also revealed a close relationship between *F. nucleatum*‐positive CRC and diet,^51^, ^75^ demonstrating the complexity of the bacteria‐cancer interacting network. The presence of *F. nucleatum* was also identified in esophageal squamous cell carcinoma (ESCC) by measuring its DNA level using qPCR.^40^ The cancer‐specific survival showed a negative correlation with the relative abundance of *F. nucleatum*, suggesting that *F. nucleatum* might be a prognostic biomarker for ESCC.^40^ Recently, high *F. nucleatum* burden exhibited a poor chemotherapeutic response in two cohorts,^52^ suggesting that *F. nucleatum* might impair the effect of chemotherapy and upregulate the drug tolerance in ESCC. Moreover, high levels of *F. nucleatum* were observed in gastric cancer tissues when compared with gastritis and intestinal metaplasia,^26^ confirming its promoting role in digestive tract carcinoma. However, during the progression of pancreatic cancer, phylum *Fusobacteria* was associated with lower pancreatic cancer risk.^27^ *F. nucleatum* harbors several membrane‐related and surface‐associated proteins, whose function is to interact with microorganisms and host cells.^53^, ^54^ This kind of interaction may lead to the pathogenic ability of *F. nucleatum* to bind and/or invade multiple cell types including oral and colonic epithelial cells.^55^, ^56^

### 2.2 *Porphyromonas gingivalis*

*Porphyromonas gingivalis* is a Gram‐negative anaerobic pathogenic bacterium involved in the destructive process of periodontitis.^57^ Due to its property of perturbing epithelial tissues and host defense mechanisms, *P. gingivalis* has recently been considered to have potential influence on the development of tumors. *P. gingivalis*, a common oral pathogenic strain, was proven to be present at OSCC sites, detected by the strong positive immunohistochemical staining by rabbit *P. gingivalis* polyclonal antibodies.^41^ As orodigestive tract is a continuous smooth passage, *P. gingivalis*, which has substantial abilities of mobility and invasion when compared with other oral bacteria, could possibly spread through the whole area and accelerate the process of in‐situ tumorigenesis. The chemotherapeutic resistance induced by colonization of *P. gingivalis* was also observed,^58^ suggesting that periodontitis was an obstacle for the treatment of oral cancer. The shift of *P. gingivalis* from the oral cavity to other areas of orodigestive tract can easily occur with the passage of food and water. It was reported almost 15 years ago that poor oral health status showed a dose‐dependent association with esophageal squamous dysplasia, a precancerous lesion of ESCC.^59^ Connection between oral bacteria and esophageal cancer has recently been confirmed through a clinical research suggesting that higher levels of *P. gingivalis* were associated with a higher incidence of ESCC.^60^ Positive intensity of immunohistochemical staining for *P. gingivalis* was much more significant in ESCC tissues compared to adjacent or normal tissues,^42^ indicating a possible relationship between *P. gingivalis* and ESCC. Median serum levels of *P. gingivalis* immunoglobulin (Ig) G and IgA were higher in ESCC patients than in controls,^43^ demonstrating that a combination of IgG and IgA could be used in the diagnosis of ESCC patients. In addition to cancers in the upper digestive tract, pancreatic cancers also possess a strong association with oral bacteria. Tooth loss, one of the most common oral pathologies, was positively linked to pancreatic cancer.^61^ Considering the fact that periodontitis is a major cause of tooth loss, further studies revealed that periodontal diseases also shared a positive relationship with risk of pancreatic cancer.^62^ Association of *P. gingivalis* with higher risk of pancreatic cancer was demonstrated by 16S rRNA sequencing of oral wash samples.^27^ Based on previous studies, serum antibody level of *P. gingivalis* could serve as a clinical biomarker for pancreatic cancer, as *P. gingivalis* serum antibody level was positively correlated with orodigestive cancer mortality.^44^ This discovery paved the way to explore the role of serum IgG and subsequently, plasma antibodies against *P. gingivalis* were confirmed to increase in patients with pancreatic cancer, while antibodies against commensal oral bacteria showed a negative correlation with pancreatic cancer risk.^45^ Infection and invasion of bacteria into the pancreas is observed in pancreatitis.^76^, ^77^ Moreover, in pancreatic abscess, a biofilm of microorganisms was also observed.^78^ Thus, the existing pathologies of pancreas might induce the accumulation of oral microorganisms and their pathological effect might elicit carcinogenesis in the pancreas. Associations between pancreatic cancer and abundance of *P. gingivalis* in oral wash samples as well as between pancreatic cancer and increased levels of antibodies against *P. gingivalis* have been proven.^27^, ^45^ Pathogens can enter the pancreas through diverse routes including blood stream, bile duct, small bowel, and reflux into the pancreatic duct.^79^, ^80^ Thus, it might be speculated that *P. gingivalis* originating in the oral cavity may spread to the cancer sites or may disturb the immune system to accelerate the development of pancreatic cancer.

### 2.3 Other anaerobic bacteria

In addition to *F. nucleatum* and *P. gingivalis*, some of the other oral anaerobic bacteria were also listed as potential carcinogens, as they have some pathogenic properties similar to those of *F. nucleatum* and *P. gingivalis*. Their clinical relationship with tumor progression has been identified using multiple methods.

The genus *Streptococcus* contains more than 100 recognized species, most of which are anaerobic and are associated with human or animal hosts.^81^ Some of them are normal inhabitants of the oral cavity, but some species exhibit high pathogenicity. Increased levels of *Streptococcus* in saliva were associated with a higher risk of gastric cancer.^28^ *Peptostreptococcus* and *Peptococcus* were highly abundant in OSCC^29^ and CRC samples.^30^ However, it was also reported that *Streptococcus pneumoniae* was associated with a lower risk of esophageal adenocarcinoma.^60^ Functions of different species of *Streptococcus* are found to be very different from one another. Many of these functions are linked to multiple oral and systemic diseases. As a predominant salivary genus, variation in its levels is proposed to be linked to abnormalities including cancers. Advances in sequencing techniques would be better equipped to explain the roles of different species of *Streptococcus* in carcinogenesis.

In addition, some other anaerobic oral bacteria with potential pathogenic properties are widely involved in tumorigenesis. High levels of *Filifactor* and *Catonella* were demonstrated in OSCC samples,^29^ while *Actinomyces* and its parent taxa up to the phylum level were significantly abundant in paired normal tissues when compared with OSCC tissues.^31^ Higher levels of *Aggregatibacter* in saliva were observed to denote a high risk of pancreatic cancer,^27^ while *Leptotrichia* was related with lower pancreatic cancer risk.^27^ A recent study that used 16S rRNA sequencing reported that *Aggregatibacter* was highly associated with throat cancer.^32^ *Prevotella* and *Prevotella 7* are considered to be associated with a lower risk of gastric cancer.^28^ *Bacteroides* was related with a high risk of throat cancer in one study,^32^ while higher abundance of order *Bacteroidales* in ESCC was also validated in a large population.^27^ *Ruminiclostridium* was shown to be related to throat cancer.^32^ Similarly, *Clostridium* sp. was also linked to OSCC.^33^ Increased levels of *Veillonella* in saliva samples were considered to predict a high risk of gastric cancer and lung cancer.^28^, ^82^ Loss of *Neisseria* in saliva was validated as a marker for pancreatic cancer.^83^ Elevated levels of *Capnocytophaga* were associated with a high risk for OSCC, while relatively lower levels of *Capnocytophaga* were observed in saliva from patient with pancreatic cancer.^83^ *Oribacterium* level was higher in the tongue coat from patients with liver carcinoma.^34^ *Leptotrichia* was more abundant in patients with pancreatic cancer in one study while its level was decreased in the saliva of patients with pancreatic cancer in another study.^27^, ^83^ *Lautropia* was also suggested to act as a marker for ESCC.

### 2.4 Aerobic bacteria

Aerobic bacteria, referred to as the bacteria whose survival is dependent on aerobic local environment, play crucial roles in the oral cavity. Unlike many anaerobic bacteria whose shared pathogenesis is linked to different types of oral diseases, most of the aerobic oral bacteria are located in the superficial areas and act as commensal bacteria to maintain oral microbial balance. Only a few aerobic bacteria have pathogenic abilities.^84^ Apart from anaerobic oral bacteria who have close relationships with various types of carcinomas possibly due to their pathogenic abilities and special vital capacity deep inside human tissues,^85^ some of the aerobic bacteria located in the oral cavity were also observed to be linked to carcinogenesis.

*Parvimonas* was highly decreased in normal oral sites,^31^ while it was highly abundant in OSCC samples.^29^ Salivary *Parvimonas* level was decreased in CRC.^29^, ^30^ *Dialister* showed high accumulation in OSCC samples.^29^ Genus *Pseudomonas* was found to be highly associated with throat cancer using 16S rRNA sequencing^32^ and its species *Pseudomonas aeruginosa* was predicted to accumulate in OSCC.^24^ In oral biofilm from central lesions of oral carcinomas, level of *Enterobacteriaceae*, which is not a common oral inhabitant, was also increased to some extent.^33^

### 2.5 Viruses

Apart from oral bacteria, viruses might also contribute to tumorigenesis. HPV is the most acknowledged virus associated with oral carcinogenesis.

Human papillomavirus infection is a major cause of cervical cancer, which is the third most prevalent cancer in women in the United States. It is also a causative factor for the development of oral cancer.^63^ Among all types of HPV, some are not involved in the pathogenesis, while others exhibit a potential for cancer development. HPV 16 and HPV 18 are involved in approximately 70% of all cervical cancers.^64^ These are indicated as high‐risk types of HPV. Recent researches have identified an increased incidence of HPV infection (approximately 50%) in head and neck squamous cell cancers.^65^, ^66^ HPV 16 was found to be the most frequent type in at least 90% of these cases.^67^ Thus, high‐risk viruses such as HPV 16 are associated with cervical cancer as well as with OSCC, suggesting a common link among different types of HPV‐related cancers. This could be explained partly by changes in sexual behaviors. High‐risk sexual behaviors such as oral and genital sex have been observed to be linked with HPV transmission through oral cavity and genital sites.^68^, ^69^, ^70^ Recent birth cohorts show an increased incidence of high‐risk sexual behaviors including practice of premarital sex, a greater average number of lifetime sexual partners, and oral sex.^71^, ^72^ Such sexual behaviors combined with some sexually transmitted diseases could partly account for the mutual communication of HPV from the genital sites to oral ones and vice versa.^73^ A recent authoritative review summarized the clinical evidence from a large cohort of oropharyngeal cancer patients and found that 72.7% of the oropharyngeal carcinomas in the twenty‐first century were HPV positive largely due to oral sexual behaviors.^74^ This conclusion from a large clinical sample strongly supports the correlation between HPV infection and oropharyngeal carcinoma.

### 2.6 Fungi and parasites

Though oral fungi and parasites are considered minorities in the oral microbial ecosystem, they are also suspected as carcinogenic candidates, as fungi and parasites located in other parts of human body have been discovered to promote tumor progression. However, little is known about the potential role of oral fungi and parasites in carcinogenesis.

Recently, pan‐pathogen array technology (PathoChip) was applied to describe the microbial biomarkers unique to human OSCC tissues. In addition to oncogenic bacteria and viruses, parasites and fungi were also detected in the array.^38^ It could be speculated that variations in the levels of oral parasites and fungi might be associated with oral carcinogenesis, but lack of adequate evidence prevents us from arriving at a definite conclusion. Further studies are necessary for better elucidation of the role of parasites and fungi in carcinogenesis.

## 3 TUMORIGENESIS‐ASSOCIATED BIOLOGICAL BEHAVIORS OF ORAL MICROBIOTA

**FIGURE 2:**
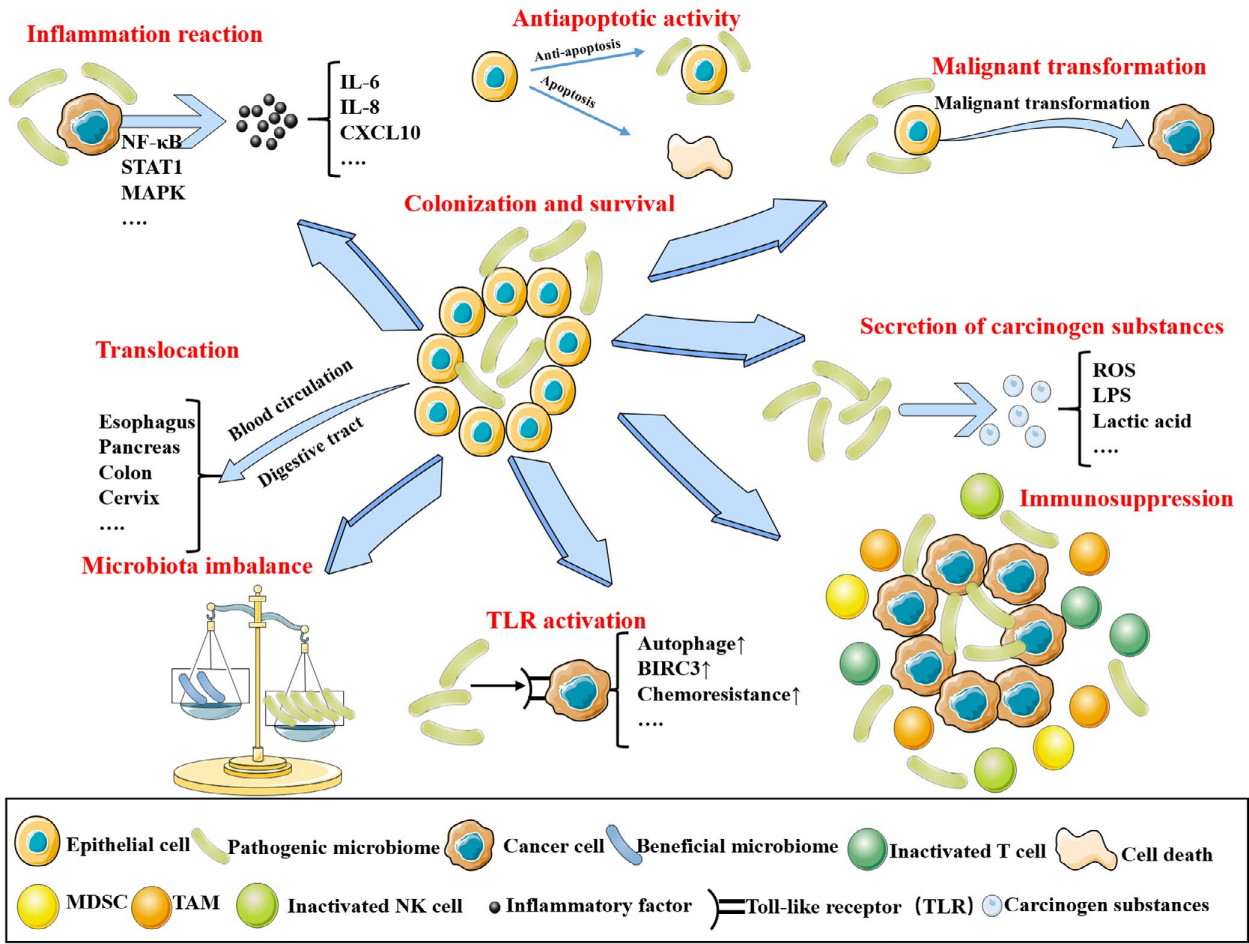
Mechanisms of the microbiota‐associated carcinogenesis. The figure depicts seven common and well‐acknowledged mechanisms for the microbiota‐associated carcinogenesis. After successful colonization and survival, pathogenic microbiota could promote the development of cancer via inflammation response, malignant transformation of epithelial cell, immunosuppression, induction of microbiota imbalance, promotion of antiapoptotic activity, and secretion of carcinogen substances. Besides in‐situ colonization, oral microbiota could translocate into other parts of human body through blood stream or digestive tract

Though ample evidence about the link between oral bacteria and cancers has emerged, little is known about how oral microbiota can influence the process of carcinogenesis. Before the direct oncogenic impact on carcinogenesis, some important tumorigenesis‐associated microbial behaviors may be observed. For oral in‐situ carcinogenesis, colonization and survival of microbiota as well as subsequent microbial dysbiosis are prerequisites, after which microbial oncogenic effect could be exerted successfully. For systemic distal carcinogenesis, translocation ability is a crucial factor in the traffic of microbiota from the oral cavity to other parts of human body (Figure 2).

### 3.1 Colonization and survival of oral microbiota inside tissues

The first step in the implementation of microbial carcinogenesis is colonization and survival of cancerous in‐situ microbiomes. Prolonged and persistent colonization by pathogenic microbiota enables further carcinogenic effect. Based on its ability to infect and colonize the basal epithelial cells of the skin or the mucosa and to use the differentiation pathway of the epithelial cells to complete its lifecycle,^86^ HPV could persistently survive in the oral cavity and in the vagina as a potential source of carcinogenesis. Traditional opinions support that other parts of the body such as the genital tract can be ideal sites for the virus to be latent, whereas the oral cavity is thought to be able to defend against the invasion by pathogens. However, it was reported recently that HPV could also infect gingival tissues.^87^, ^88^ This could be explained by the fact that some high‐risk factors such as human immunodeficiency virus infection, smoking, tobacco use, and immunosuppression may damage the defense mechanism of the oral cavity.^89^ Moreover, periodontitis might also favor the persistence of HPV in the oral cavity due to destruction of the integrity of mucosal barrier.^90^ Differentiation pathway of the epithelial cells is necessary for HPV to complete its lifecycle,^86^ as replication of viral DNA occurs only within the basal layers of the epithelial cells that are destined to undergo maturity and senescence.^91^ Often, the movement of basal epithelial cells during wound healing is necessary for the lifecycle and the spread of viral particles, which takes approximately 2‐3 weeks.^92^ *F. nucleatum* harbors several membrane occupation and recognition nexus repeat‐containing proteins, whose function is to interact with the microorganisms and the host cells.^53^, ^54^ Such an interaction acts as a groundwork for the pathogenic ability of *F. nucleatum* to bind and/or invade multiple cell types including oral and colonic epithelial cells.^55^, ^56^ The localization of *F. nucleatum* to cancerous tissues is mainly attributed to fusobacterial protein Fap2, which can recognize and bind to Gal/GalNAc, a membrane protein overexpressed in CRC cells.^93^ The Fap2‐dependent‐specific binding mechanism instead of normal endocytosis suggests a unique interaction of *F. nucleatum* with CRC cells. Moreover, the unique FadA adhesin of *F. nucleatum* could help in the binding to E‐cadherin and activation of β‐catenin signaling pathway, thus regulating the inflammatory and oncogenic responses.^94^ The variations in the survival rate of *P. gingivalis* in acidic conditions at different pH values in combination with the distribution of *P. gingivalis* in different regions of the upper digestive tract could partly play a role in the colonization of *P. gingivalis* in the upper digestive tract.^95^ This finding supports the causative role of *P. gingivalis* in ESCC, as it clarifies the possibility for the colonization of *P. gingivalis*.

### 3.2 Microbial dysbiosis in the oral cavity

Various commensal oral bacteria have a negative correlation with the risk of pancreatic cancer. It is a well‐known fact that oral diseases commonly originate from changes in the balance of microbial ecology,^96^, ^97^ which means decrease in the number of beneficial bacteria and increase in the number of harmful ones. Microbial dysbiosis has been widely linked to inflammation‐associated CRC,^98^ which is a new insight focusing not only on the effect of specific pathogens but also on the imbalance of the entire microbial ecosystem. Many types of oral bacteria with a change in the diversity of their species may lead to functional alteration of the oral ecosystem during carcinogenesis. Abnormality in the relative microbial levels is a common sign suggestive of oral microbial imbalance. Abnormal changes in the oral microbial richness have been observed to be linked to different types of cancers. A recent cross‐cohort meta‐analysis revealed a greater richness of oral microbial species in CRC tissues, indicating an influx of bacteria originating in the oral cavity.^30^ In contrast, lower richness and diversity were observed in the salivary samples of patients with acute myelocytic leukemia when compared with healthy controls.^99^ Microbial diversity of the tongue coat in liver cancer patients also showed an obvious increase when compared with healthy population.^34^ Tongue coat, salivary samples, and subgingival samples collected from patients with gastric cancer revealed a reduced microbial diversity in the tongue coat of patients and surprisingly, an increased microbial diversity in the salivary and the subgingival samples.^48^, ^75^ Recently, microbial diversity of saliva was shown to decrease with throat cancer progression.^32^ Inflammation and immunodeficiency are the two most noticeable outcomes of dysbiosis.^100^ They are also among the crucial mechanisms of carcinogenesis. Oral cavity serves as an open environment, which is under the influence of many external factors such as smoking and drinking. Recent studies identified dysbiosis of oral cavity caused by excessive smoking and drinking.^101^, ^102^ Consistent with these observations, smoking and drinking are risk factors for the development of many types of cancers. Hence, it might be speculated that oral microbial imbalance associated with alterations caused by external factors is another mechanism for tumorigenesis. It could also be speculated that the imbalance of microbiota reflects a damaged immune function, imposing a pathogenic influence on carcinogenesis globally.

### 3.3 Microbial translocation from the oral cavity to other parts of the body

The translocation ability of oral microbiota is important for the development of systemic cancers associated with oral infection in addition to many oral bacterial diseases. It is speculated that blood circulation is a probable pathway, as periodontal bacteria always migrate to other regions such as atherosclerotic lesions^103^ and brains of patients with Alzheimer's disease^104^ through blood vessels. Orodigestive tract is also a plausible passage, as movement of bacteria could cooperate with the flow of food and fluid into the digestive system fluently. This finding was confirmed by the presence of oral bacteria in distal esophagus.^105^ Bleeding is a common complication during periodontal therapy. Through this route, oral microbiota could enter the blood circulation and colonize the regions suitable for their survival. Moreover, some mobile oral bacteria could move toward distant regions through the airway and the digestive tract during drinking or eating. Thus, there is ample evidence to speculate that after translocation, oral microbiota could contribute to cancer development in regions other than the oral cavity. Indeed, the microbial translocation ability may be an initial mechanism for the development of distant carcinomas.

## 4 MECHANISMS UNDERLYING THE ROLE OF ORAL MICROBIOTA IN CARCINOGENESIS

Evidently, the role of oral microbiota is a double‐edged sword in carcinogenesis, as both antitumor and pro‐tumor microbial functions have been discovered. Some prevalent opinions about oral microbial oncogenic mechanisms have been summarized and discussed in the following subsections. However, further exploration is still needed in this field.

### 4.1 Excessive inflammatory reaction in response to microbiota

Though moderate inflammatory reaction is protective against tumorigenesis, excessive inflammatory response is reportedly a primary contributor to carcinogenesis in many types of cancers.^106^, ^107^, ^108^ Invasion of pathogenic microbiota could also elicit various inflammatory diseases.^109^ Hence, the carcinogenic effect of the microbiota is partly due to their induction of inflammation. Many oral microorganisms associated with carcinogenesis are pathogenic bacteria or conditional pathogenic bacteria such as *Porphyromonas*, *Prevotella*, and *Fusobacterium*, which could induce chronic inflammatory reaction. Production of some well‐known inflammatory mediators and effect molecules is highly elevated due to the imbalance of oral microbiota. Local concentrations of cytokines such as interleukin‐1β (IL‐1β), interleukin‐6 (IL‐6) as well as matrix metalloproteinases (MMPs) are highly elevated, as observed in the pathogenesis of periodontitis.^110^ IL‐1β acts as a motivator for the progress of inflammation due to its ability to release prostaglandins, tumor necrosis factor, and IL‐6.^111^ Moreover, IL‐1β itself has been considered to have a great potential to promote tumor metastasis^112^ and malignant transformation in OSCC.^113^ As a principal downstream signaling molecule, IL‐6 is also involved in cancer initiation, promotion, progression, and metastasis.^114^ The translational value of IL‐6 as a therapeutic target in cancer treatment also underlines its importance in the inflammation‐associated cancer pathogenesis.^115^ Low levels of bacteria‐induced tumor necrosis factor are also reported to be associated with tumor promotion.^116^ Microbiota‐induced or IL‐1β‐induced overexpression of MMPs is a crucial factor in tumor migration, as degradation of extracellular matrix and loss of cell‐cell or cell‐matrix adhesion caused by MMPs might contribute considerably to tumor invasion and tumor spread.^117^ Some inflammatory signaling pathways commonly activated by these cytokines such as nuclear factor‐κB (NF‐κB), Wnt, and JAK‐STAT3 cascades are also associated with carcinogenesis from genetic perspective, which might explain the influence of microbiota on cancer development to some extent. Analysis of microarray data has revealed changes in several hub genes such as IL‐6, signal transducer and activator of transcription 1, and C‐X‐C motif ligand 10 after *P. gingivalis* infection. Most of these could act as upstream regulators of carcinogenesis.^32^ Using molecular biological technology, some explanations for this phenomenon have emerged. The virulence of bacteria is mainly due to the inflammatory response of invaded tissues, which could also account for the progression of many types of cancers. Interleukin‐8 (IL‐8) is a key factor in inflammation that has been found to participate in *P. gingivalis*‐induced oral carcinogenesis.^93^ Similarly, inflammation‐associated classical cascades, such as NF‐κB, mitogen‐activated protein kinases, programmed death‐ligand 1 (B7‐H1), and programmed death‐ligand 2 (B7‐DC), have been identified to intervene in the malignancy of oral cancer cells.^93^, ^118^ A recent study suggested that blockade of immune checkpoints could effectively diminish the carcinogenic influence of *P. gingivalis* in ESCC.^119^ *F. nucleatum* can also induce high‐level expression of some pro‐inflammatory cytokines including IL‐6 and IL‐8,^31^ which are key promoters in inflammation‐related diseases.

### 4.2 Pathogen‐induced immunosuppression of host

Immunosuppression is also known to play an important role in the development of many types of cancers.^120^ Many pathogenic microbes are involved in the suppression of host immunological function. Microbiota‐associated immunosuppression is an important contributor to carcinogenesis. Complicated techniques of immune evasion allow HPV to remain undetected for a long time and to deploy its oncogenic effect persistently.^121^ Proteins E6 and E7 play a significant roles in this process. E6 reduces surface expression of CDH1,^122^ a key component for antigen presentation, and the expression of toll‐like receptor 9,^123^ a recognition receptor for viral pathogens. E7 decreases the expression of transporter associated with antigen processing 1, preventing the activation of T lymphocytes.^124^ Downregulation of proinflammatory cytokines such as tumor necrosis factor α (TNF‐α) and upregulation of anti‐inflammatory cytokines such interleukin‐10 also take place during infection.^125^ *P. gingivalis* has been proven to suppress the immune system by invasion of host cells and disruption of immune‐related signaling pathways,^126^, ^127^ which might influence the malignant process of pancreatic cancer. The intra‐tumoral bacteria also play a key role in this process. By recruitment of tumor‐infiltrating immune cells, *F. nucleatum* creates a proinflammatory tumor‐associated environment consisting of many myeloid‐derived suppressor cells and tumor suppressing immune cells, which is conducive for neoplasia.^128^ M2 polarization of macrophages always contributes substantially to tumor‐associated environment. This phenomenon is also observed in CRC associated with *F. nucleatum* infection.^129^ Another pathway exploited by *F. nucleatum* is inhibition of natural killer cells and various T lymphocytes via Fap2 binding to T‐cell immunoreceptor with Ig and ITIM domains in these cells, affecting antitumor immune activities.^130^

### 4.3 Induction of malignant transformation

After stable colonization and survival, microbial effect on the epithelial cells to promote their malignant transformation is crucial for the initial phase of carcinogenesis. After initial colonization, some key proteins derived from HPV launch the malignant transformation of epithelial cells. Among these, the most important proteins are E6 and E7, which have the ability to prolong cell cycle, activate cellular proliferation, and prevent apoptosis.^131^ Due to the impact of E6 and E7, infected cells cease to undergo apoptosis and remain involved in persistent cell cycle. This ability is explained by the interaction between RB1, RBL1, and E7^132^, ^133^ as well as by degradation of TP53 induced by E6.^134^ With accumulation of genetic alterations, transformation from normal cell to invasive cancer cell finally takes place. Prolonged and repetitive exposure to *P. gingivalis* may be implicated in the malignant transformation of normal oral epithelial cells,^135^ suggesting that severe periodontitis might contribute substantially to oral carcinogenesis. Furthermore, acquisition of cancer stem cell properties via *P. gingivalis* infection is thought to play a role in enhancing the aggressiveness of oral cancer cells.^82^ Based on the finding that *F. nucleatum* could interact with intestinal epithelium as well as with oral epithelium, studies have identified that *F. nucleatum* could initiate the host molecules of normal oral epithelium and promote the predisposition to malignant transformation through epithelial‐mesenchymal transition.^136^ Many oral anaerobic bacteria share a similar ability to interact with epithelial cells and long‐term exposure to these bacteria might result in epithelial malignant transformation. Thus, interactions between oral microbiota and normal epithelial cells could result in a great number of changes in gene expression including changes in mRNAs, miRNAs, and LncRNAs. Many of these genetic alterations such as P53 downregulation are associated with malignant transformation. Hence, along with the positive role of oral microbiota in promoting cancer development, it is speculated that oral microbiota can also guide the malignant transformation of normal epithelium to initiate tumorigenesis. If this phenomenon is verified in the future, oral microbiota might be regarded as a cancer‐initiating factor rather than just a risk factor. Thus, greater attention ought to be paid to the association between oral microbiota and cancer.

### 4.4 Promotion of antiapoptotic activity

The antiapoptotic ability of cancer cells is a malignant feature that could lead to long‐term proliferation and chemoresistance of carcinoma. *P. gingivalis*‐induced promotion of antiapoptotic activity is the best example to explain the role of oral microbiota in this phenomenon. Reportedly, *P. gingivalis* induces changes in the intrinsic mitochondrial apoptotic activity via JAK1/AKT/STAT3 pathway.^137^, ^138^ Phosphorylation of BAD and enhancement of the ratio of BCL2 to BAX caused by *P. gingivalis* infection could significantly reduce the apoptotic activity of epithelial cells.^139^ Moreover, secretion of antiapoptotic enzyme nucleoside diphosphate kinase by this bacteria cleaves adenosine triphosphate and prevents the activation of P2X7, decreasing the apoptotic activity modulated by these molecules. Activation of toll‐like receptor 4 by *F. nucleatum* results in upregulation of autophagy and downregulation of apoptosis, leading to a chemoresistance phenotype.^140^ Since many of these cancer‐associated oral microbes share the same pathogenic properties and could activate or inactivate similar receptors or signaling pathways, it could be speculated that many microbiota‐associated cancer phenotypes such as chemoresistance might be explained partly by the promotion of antiapoptotic activity.

### 4.5 Production of carcinogenic substances

A recent study has reported a significant carcinogenic role of a metabolic genetic toxic substance named colibactin secreted by *Escherichia coli*.^141^ This great discovery has ignited interest in the microbiota‐produced carcinogens. Similar to intestinal microbiota, substances produced by oral microbiota might also be associated with carcinogenesis. Reactive oxygen species are cell metabolic products generated during microbiota‐induced inflammation.^142^ Their contribution to cancer pathogenesis has been verified in various processes such as cellular transformation, tumor survival, invasion, angiogenesis, and metastasis.^143^ Similarly, reactive nitrogen species might also exhibit similar potential. Some known oral peroxigenic bacteria including *Streptococcus oralis*, *S. mitis*, *S. sanguinis*, *S. gordonii*, *S. oligofermentans*, *Lactobacillus fermentum*, *L. jensenii*, *L. acidophilus*, *L. minutus*, and *Bifidobacterium adolescentis* ^144^ might pose carcinogenic influence. Lipopolysaccharide (LPS) is a pathogenic substance commonly shared by many anaerobic oral bacteria. Its ability of activating inflammatory process is widely linked to inflammation‐associated cancer pathogenesis. Many cancer‐associated cytokines such as IL‐1β, IL‐6, and TNF‐α are elevated due to LPS stimulus during oral infection.^145^ Similarly, volatile sulfur compounds produced by some Gram‐negative oral bacteria exhibit toxic and inflammation‐inducing effect and might play a role in carcinogenesis.^146^, ^147^ Gingipains produced by *P. gingivalis* are major pathogenic substances whose pathologic features have been reported in periodontitis and Alzheimer's disease. They might also be linked with tumorigenesis, as their ability to activate inflammatory signaling and MMP9 might promote tumor migration to some extent.^148^ Many oral bacteria such as genera *Lactobacillus*, *Lactococcus*, *Bifidobacterium*, *Streptococcus*, *Leuconostoc*, and *Pediococcus* have the potential to produce lactic acid.^149^ Studies have identified the role of lactic acid in the immunosuppression of cancer sites, making it a useful therapeutic target.^150^, ^151^ On the other hand, overproduction of acidic substances could result in low pH and hypoxic microenvironment, which is greatly suitable for tumor metastasis.^152^ Moreover, many taxa isolated from oral cancer samples include aciduric species,^153^ demonstrating the link between oral microbiota‐derived acidic products and carcinoma (Table 3).

**TABLE 3:** Impact of oral microbial carcinogen on carcinogenesis

Carcinogen	Source	Effect	References
ROS, RNS	*Streptococcus oralis*, *S. mitis*, *S. sanguinis*, *S. gordonii*, *S. oligofermentans*, *Lactobacillus fermentum*, *L. jensenii*, *L. acidophilus*, *L. minutus*, and *Bifidobacterium adolescentis*	Promotion of cellular transformation, tumor survival, invasion, angiogenesis, and metastasis	142, 143, 144
VSCs	*Porphyromonas gingivalis*, *Prevotella intermedia*, *Aggregatibacter actinomycetemcomitans*, *Fusobacterium nucleatum*	Induction of inflammatory reaction	146, 147
LPS	Gram negative oral bacteria	Induction of inflammatory reaction	145
Gingipains	*P. gingivalis*	Activation of inflammatory signaling Induction of MMP9	148
Lactic acid	*Lactobacillus*, *Lactococcus*, *Bifidobacterium*, *Streptococcus*, *Leuconostoc Pediococcus*	Promotion of immunosuppression Maintaining of hypoxic microenvironment Promotion of tumor metastasis	149, 150, 151, 152, 153

Although multiple pathways of microbiota‐induced carcinogenesis have been reported, the most prevalent and well‐studied mechanisms include direct influence on cancer cells and indirect effect via immune regulation. The existing observations could shed more light on other pathways that remain to be discovered.

As strong clinical correlation has been discovered between microbiota and carcinogenesis, studies about pathogenic mechanisms of microbiota‐induced cancer are making great progress currently. However, more details are needed to clarify the basic biology of cancer‐associated oral microbiota, the interaction between microbiota and host cells, and the mechanism used by oral microbiota to influence other microorganisms and to shape microenvironments.

## 5 CONCLUSIONS

The concept of microbiota‐associated cancer is currently a hot topic with great attention from microbiome researchers and tumor researchers. Oral cavity serves as one of the largest microbial storage in human body and the microbial variations in the oral cavity might be highly linked with malignancies. From clinical information and basic research, some evidence about oral microbiota and carcinogenesis has been discovered. However, further studies are still necessary for an accurate and complete understanding of this relationship.

The abundance of oral bacteria specifically associated with cancer appears to be a clinical biomarker to some extent, although further exploration is still necessary. Oral health detection indices could be used to predict multiple global health problems, as the oral cavity could act as a window toward deeper areas of human body. If this clinical phenomenon is confirmed, early prediction of some types of cancers using microbial detection might be a crucial breakthrough. The testing samples of oral microbiome vary among salivary samples, tissue samples, fecal samples, and blood samples. Microbial RNA expression level, protein level, or specific serum antibody level might be selected as detection index. However, selecting the index that could accurately predict cancer‐associated microbial variation needs further clinical experiments. Due to the multiformity and accuracy of microbial detection techniques, oral microbial status has a great potential to be a biomarker for related cancers and to help arrive at precise diagnosis and prediction.

Once the mechanisms of microbial carcinogenesis are fully elucidated, their application in therapy might be explored. Currently, combination therapy is a well‐accepted cancer treatment due to the complexity associated with cancer formation and development. As microbial factors could contribute to cancer development and chemoresistance, the use of antimicrobial treatment might be included in the combination strategy. Removal of pathogenic microbiota might overcome the negative influence of infectious factors and contribute to enhancing the effect of chemotherapy. Further clinical studies are necessary to prove whether anti‐pathogen therapy could be applied to clinical treatment. If anti‐pathogen treatment could be confirmed as a useful approach in clinical therapy, survival and prognosis of cancer patients might be improved significantly.

## CONFLICT OF INTEREST

The authors declare that they have no competing interests.

## AUTHOR CONTRIBUTION

Jiwei Sun and Qingming Tang contributed equally to this review and should be considered co‐first author. Conceptualizations, Jiwei Sun and Qingming Tang; literature collecting and preparation, Jiwei Sun and Qingming Tang; writing‐original draft preparation, Jiwei Sun and Qingming Tang; writing‐review and editing, Jiwei Sun, Qingming Tang, Shaoling Yu, Mengru Xie, Yanling Xie, and Guangjin Chen; Supervision, Lili Chen.

## ETHICS APPROVAL

This study was approved by Tongji Medical College, Huazhong University of Science and Technology.

## Data Availability

The data used to support the findings of this study are available from the corresponding author upon request.
